# Intelligence

**Published:** 1833-07

**Authors:** 


### INTELLIGENCE.

### THE HUNTERIAN ORATION FOR 1833, BY JOHN HOWSHIP, ESQ.

We confess we perform a too tardy act of justice in now noticing the well-conceived, and equally well-delivered, oration of Mr. Howship. With some few exceptions, these annual exhibitions at the College have not been particularly attractive. It is almost impossible, indeed, that each year can produce, even from different hands, any very interesting dissertation upon one topic; and, of course, the more frequently these orations are repeated, the greater will be the ingenuity required to offer any original comments either upon the splendid talents of Mr. Hunter, or his celebrated museum.

Mr. Howship commenced by giving a neat and rapid sketch of the progress of medicine and surgery from the most remote ages of antiquity; and, in passing down to nearer periods, he neatly and eloquently descanted upon the names of Michael Angelo, Leonardo Da Vinci, and Raphael, who added to their well-known talent for painting the merit of being good practical anatomists. Here Mr. Howship embraced the opportunity of enforcing the advantages which result to the anatomical student from a knowledge of the principles of drawing; and he expressed a hope that, "as the Royal Academy of Painting has its professor of anatomy, we may one day see the Royal College of Surgeons have also its Professor of Design." Due honour was paid by the orator to the names of Harvey, Wiseman, Haller, and Hunter. A rapid sketch was given of the contents of Mr. Hunter's museum; and to Mr. Abernethy was assigned the merit of being "the most eloquent, most ingenious, and at the same time the most faithful, commentator" of that great man. The loss which science has sustained in the death of Baron Cuvier was feelingly and appropriately lamented. Mr. Howship mentioned him as the "ornament of France, the admiration of Europe, and the envy of the civilized world."—The oration was delivered extempore; and, at its conclusion, Mr. Howship was loudly and repeatedly cheered.

---

*Apothecaries' Hall; 31st May, 1833.*

Sir: I am directed by the master and wardens of the Society of Apothecaries to transmit to you the enclosed copies of a Memorial to the Secretary of State, on the subject of the Act of Parliament for regulating the Practice of Apothecaries, and of a Reply to the Statement made by the Royal College of Surgeons of Edinburgh, in support of a petition presented by them to both Houses of Parliament. I am, sir, your obedient, humble servant,

Edmund Bacot, *Clerk of the Society.*

John North, Esq.

To the Right Honourable Lord Viscount Melbourne, his Majesty's Principal Secretary of State for the Home Department.

*The Memorial of the Master, Wardens, and Society of Apothecaries of the City of London,*

Sheweth: That the Act for regulating the Practice of Apothecaries in England and Wales was passed in the year 1815, with the full concurrence of the medical profession in England, and was loudly called for by the then state of medical knowledge.

That the Act has been impartially and honestly administered, and the Court of Examiners of the Society, by their attention to the medical education of students, and by requiring from time to time a progressively increasing course of study, now equal to the medical education required by any college in Great Britain, have most essentially improved the schools of medicine in England, as well as the state of medical science in general; and the general practitioners of medicine throughout England and Wales, who have been educated in conformity with the regulations of the Court of Examiners, are now fully competent to all the duties of their profession, and are entitled to, and actually possess, the confidence of the public.

That the power of examining persons who are about to commence practice as apothecaries can in nowise be considered a "monopoly;" inasmuch as the persons who examine the candidates for certificates of qualification have not the most remote interest in, or connexion with, any school of medicine, nor can any teacher in any branch of medical science become a member of the Court of Examiners.

That the clause in the Act of Parliament, whereby an apprenticeship of five years is required to be served by persons before they can be examined, was not proposed by the Society of Apothecaries, but was introduced into the Act in the House of Lords. But the Society beg to remark, that a preliminary education, in the nature of an apprenticeship, is absolutely necessary, as well to lay the foundation for future acquirements, as for the moral control of students from the age of sixteen to twenty-one; which control cannot be effectually exercised at any medical school or hospital.

That those persons who state that an apprenticeship is highly injurious to medical education, must be very ill informed upon that subject; for it is very well known that an apprentice has the opportunity of acquiring most valuable professional information from a suitable course of reading, under the direction of his master, and from seeing cases which occur in his practice.

That attendance on the physician's practice at an hospital does not afford to a student the whole of the instruction in practical medicine of which he stands in need, inasmuch as one half of the deaths which annually take place are of children under five years of age, and of the aged above seventy, which cases are very rarely received within the wards of an hospital, but are attended almost exclusively by general practitioners; consequently, coming under the observation of their apprentices.

That the different colleges and corporate bodies in Scotland, who are seeking to obtain an alteration in the Act of Parliament for regulating the Practice of Apothecaries in England and Wales, by rendering the diploma of those bodies equivalent to the certificate of the Court of Examiners of the Society, are endeavouring to obtain for their licentiates a more extensive privilege in England than their diploma confers in Scotland; inasmuch as the diplomas of the University and of the Royal College of Surgeons in Edinburgh do not confer any right on the holders of them to practise as general practitioners in Glasgow, or within the counties of Lanark, Renfrew, Ayr, and Dumbarton, over which the jurisdiction of the Faculty of Physicians and Surgeons of Glasgow extends; nor does the diploma of the Faculty of Physicians and Surgeons of Glasgow confer any right within the jurisdiction of the College of Surgeons of Edinburgh; nor can a person having a diploma or certificate from any college in England, or from the Society of Apothecaries, practise in Scotland without undergoing the examination, and obtaining the diploma, of the Scotch colleges.

That numerous instances have occurred (several within the last twelve months) of graduates and members of Scotch universities and colleges having been rejected by the Court of Examiners of the Society, in consequence of their having been found incompetent to practise as apothecaries.

That, should the prayer of the petitions presented to Parliament by the Scotch universities and colleges be granted, one effect thereof would be to authorise those persons to practise immediately, who have, on examination, been found incompetent to do so without further professional study.

That the establishment of several different tribunals would give rise to very mischievous inequality of examination, and tend progressively to great laxity therein, and to lower the standard of the required attainments; more especially wherever it may happen, as it now does, at the different universities in Scotland, that the teachers are also public examiners.

That the admission of persons to practise as apothecaries who have not received certificates from the Court of Examiners of the Society, and been announced in the list annually published in conformity with the Act, will deprive the public and the Society of the means of ascertaining the illegal practitioner, and will thereby give an opportunity for ignorant and incompetent individuals, to whom the health of the public cannot be safely intrusted, to commence and continue in practice, in defiance of the Act of Parliament.

That the sums of money received by the Society of Apothecaries for certificates of qualification are not more than sufficient to defray the necessary expenses of enforcing the law against incompetent and unqualified practitioners, and the other necessary expenses of administering the Act; and that no part whatever of the sums so received has been, or is, appropriated to the private purposes of the Society.

That there are now in England numerous schools of medicine, all of which (with the exception of a few in London) have been established since the passing of the Act of Parliament, at great expense to the parties who have established them.

That one effect in the alteration of the law sought for by the Scotch universities and colleges will necessarily be to prove a serious injury to all the schools of medicine in England.

That at these schools, including those in London, there are now attending nearly 1300 students, exclusive of nearly 2000 apprentices in other parts of the kingdom.

That 6498 persons have been examined by the Court of Examiners of the Society since the passing of the Act in 1815, to 5769 of whom certificates have been granted; and the number of general practitioners in England and Wales is now upwards of 10,000 persons.

Your memorialists therefore beg to draw your lordship's serious attention to these circumstances, trusting that due consideration will be given to a subject of such high importance to the interests of the profession and the public, and that an opportunity will be afforded to the Society to show, as they are prepared to do, that the Act of Parliament has been properly and efficiently administered, and to refute any statement which has or may be made against the Society, and to show that all the benefits which the Act of Parliament was intended to afford to the public have been fully attained.

By order, (Signed) Edmund Bacot,
*8th May*, 1833. *Clerk to the Society.*

---

A Reply *to the* Statement *in support of a* Petition *of the* Royal College *of* Surgeons *of* Edinburgh.

The Society of Apothecaries of London beg to solicit attention to a Reply to the Statement made by the Royal College of Surgeons of Edinburgh, in support of a petition presented by them to the two Houses of Parliament.

The object which the college of Edinburgh has in view is, to obtain for itself, and for the other medical authorities in Scotland, the power of authorizing all persons to practise as apothecaries in England and Wales, who already have, or hereafter may obtain, degrees or licences from the Scottish colleges, which the Act of Parliament for better regulating the Practice of Apothecaries, passed in the 55th year of his Majesty Geo. III. 1815, does not now permit them to do. Among the causes of complaint stated by the college, the following appear to be the principal:

1st, The college state "that the Society of Apothecaries, *without their knowledge*, applied for and obtained an Act of Parliament, by which all persons are prohibited, under certain penalties, from practising as apothecaries in England and Wales, unless they shall be licensed by the Society.

2d, "That the Society of Apothecaries so interpret the 15th clause of the said Act relating to apprenticeship, as entirely to exclude from examination, and licence, all medical students who have received their education in Scotland.

3d, "That the power possessed by the society is a 'monopoly;' that it is unjust to the licentiates of the Royal College of Edinburgh, as well as to others, who possess testimonials or diplomas from any of the different public bodies intrusted with the superintendence of medical education in these kingdoms; that it excludes from practice in England and Wales, persons whose certificates of qualification are much more ample than those possessed by the licentiates of the Society of Apothecaries, and whose knowledge and acquirements are above all exception; and that it is injurious to the interests of the community."

In reference to the first complaint of the college, "*That the Act was applied for and obtained without their knowledge.*" It is presumed that the college, in stating that the society applied for and obtained the Act of Parliament *without their knowledge*, intend only to represent that the society did not give them official notice of what was passing in Parliament, since it is impossible to imagine that the college could either be inattentive to, or ignorant of, a matter which excited such general and deep interest throughout the whole medical profession in England. For more than three years prior to the passing of the Act, *Medical Reform* was under discussion in almost every city, town, and village in England; numerous district associations were formed for the promotion of that object, and, in London, a central committee, consisting of the most eminent general practitioners, was appointed, among whom were many natives of Scotland. The district associations held frequent correspondence with the central committee; and, for the more effectually promoting the object which all had in view, deputies were sent from the country to communicate personally with, and assist, the London committee. The result of these conferences was, that Parliament was applied to, to create a superintending body, who should be empowered to examine candidates and grant certificates of qualification, without which no persons should be allowed to practise as apothecaries in England and Wales. The government and legislature, however, objected to grant a controlling power to any new authority, considering that the execution of the Act ought to be confided to one of the existing chartered bodies; and eventually, the Society of Apothecaries was considered by all parties to be the most proper body for this purpose, and they, in conjunction with the Association of Apothecaries, prepared a Bill, which was introduced into Parliament, with the full assent and concurrence of the Royal College of Physicians, and the sanction of his Majesty's government. This Bill, after considerable discussion, was passed by both Houses of Parliament, and received the Royal assent.

The colleges of Scotland have each their own several and separate jurisdictions, over which they exercise, by virtue of their charters, an independent government and control; they have the power of prescribing the manner and duration of the studies of persons who intend to apply to them for licence to practise, and they have the power of granting or refusing such licence, and of preventing all other persons from practising within their several jurisdictions. The College of Surgeons of Edinburgh is a chartered corporation, and within the limits of their charter, the entire government of the surgeon-apothecary's practice is confided to the college. "The Faculty of Physicians and Surgeons of Glasgow is a corporate body, erected by charter in 1599, confirmed by Act of Parliament, with power to call before it and examine all practitioners in medicine, surgery, and pharmacy, (who have not obtained a medical degree,) within the counties of Lanark, Renfrew, Ayr, and Dumbarton, and to license or interdict from practice, as may appear to them proper."*

Neither in Edinburgh nor in Glasgow can any person practise as a surgeon-apothecary, unless, in Edinburgh, he is a fellow of the college, or in Glasgow, unless he is a member of the faculty there; and so jealous are these colleges of their respective privileges, that the college of Edinburgh will not suffer a member of the Glasgow faculty to practise in Edinburgh, nor will the Glasgow faculty suffer a fellow of the college of Edinburgh to practise in Glasgow. Nor will either of these corporations permit their own licentiates to practise in Edinburgh itself, or in Glasgow, but confine the entire practice in these towns to such persons as are fellows or members.

With these general or local privileges the medical authorities of England have never at any time attempted to interfere. In claiming therefore for themselves the power of acting as circumstances may direct, with perfect independence of the Scottish colleges, the society claims no more for itself in England, than those institutions possess in Scotland.

The society contend that the Scottish colleges have no right to interfere in any manner with any enactments or regulations which the constituted medical authorities of England may deem essential to their own government, or for the welfare of the public, so long as they confine themselves to this division of the island, and do not make any attempt on the provinces of others.

Another complaint of the College of Surgeons of Edinburgh is, "That the society so interpret that clause of the Act which relates to apprenticeship, as entirely to exclude from examination and licence all medical students who have received their education in Scotland."

Had the College of Surgeons of Edinburgh made application to the Society of Apothecaries respecting the interpretation which they put on this clause, they assuredly would not have put forth a statement so fallacious as that which they have unadvisedly circulated.

The words of the Act are, "Provided always, and be it enacted, that no person shall be admitted to any such examination for a certificate to practise as an apothecary, unless he shall have served an apprenticeship of not less than five years to an apothecary." In their interpretation of this clause, the society have not confined themselves to the narrow limits ascribed to them by the college; but have, on the contrary, put upon it the most liberal construction in their power, by considering that every candidate who has been an apprentice for the length of time directed by the Act, is entitled to be examined, provided the person to whom he was an apprentice was legally qualified to practise as an apothecary according to the laws in force in *that kingdom or particular district in which he re-*

* Extract from a Report of the Faculty of Physicians and Surgeons of Glasgow, dated August 5, 1806, and signed Robert Freer, Convener.

*sided:* and in accordance with this interpretation, hundreds of candidates have been admitted to examination who have served their apprenticeships in Scotland and Ireland, as well as many from America and the British colonies. It is true, indeed, that the society do not admit candidates to be examined who have served apprenticeships in England or Wales, unless the apothecary, to whom they were apprenticed, was himself legally qualified by the law of England to practise; but the society have always admitted candidates to examination who have served their apprenticeships in Scotland, provided the master was legally qualified to practise according to the laws of that country.

It may be as well to take this opportunity of referring to a paragraph of the published statement of the college, in order to relieve the society from the imputation of having been guilty of a gross absurdity. The paragraph is this:

"To obviate the glaring injustice of this provision of the Apothecaries' Act, (viz. the necessity of apprenticeship to an apothecary), there was introduced into a Bill to amend and explain it, which was passed in the session of 1825, a clause authorizing the Court of Examiners to examine apprentices to surgeons. But, as the duration of that Bill was limited to a single year, and has not since been renewed, the provision of the original Act respecting apprenticeships has again come into force."

The facts are, that the Bill here alluded to was prepared by the society, and was introduced to the House of Commons by the present Lord High Chancellor of England, and, having passed that house, was sent to the House of Peers. The Scottish universities and colleges then attempted to procure for their graduates and licentiates the same privileges which are now sought to be obtained; and, failing to accomplish this purpose, the result was, that the duration of the Bill was limited to one year; and thus was a Bill, which was intended by the society to remove disabilities, and to amend acknowledged defects in the law, defeated. Let not the College of Surgeons of Edinburgh, then, impute to the society as a fault, that which was the decision of the House of Lords.

That opinions adverse to apprenticeships (of whatever duration) are entertained, and that such opinions are readily received among youth, and by persons unacquainted with the subject, the society have been for a long time fully aware; but they were not prepared to expect such sweeping denunciations against this method of preparatory education, as the Edinburgh college have now put forth in their statement, and which, after the well-known and recently recorded opinion of that body on the contrary side of the question, have excited no small degree of astonishment.

This recorded opinion of the college is to be found in their "Regulations to be observed by candidates previously to their being taken upon trial for obtaining diplomas from the Royal College of Surgeons of Edinburgh." As this preface to the regulations of the college has not been circulated with their published statement, the society insert an extract from it, accompanied by an extract from the printed preface to the regulations of the Court of Examiners of the society; thereby affording to the public an opportunity of learning what the opinions of the college recently were, and in what respects the College of Surgeons of Edinburgh and the Society of Apothecaries agree as to the preparatory qualifications necessary for a medical student, and as to the best course of pursuing his medical education.

COLLEGE OF SURGEONS OF EDINBURGH.

*Extract from the Preface to their Regulations.*

While they thus recommend, in the strongest manner, an attention to these preliminary branches of instruction, Latin, Greek, and modern languages, and of the elements of mathematics and natural philosophy, as the best preparation for their entering upon a course of medical and surgical education, the college wish to remind the public, that

SOCIETY OF APOTHECARIES OF LONDON.

*Extract from the Preface to their Regulations.*

Before the student enters upon his professional studies, it is indispensably necessary that he should have received a classical education, as, in addition to the advantages which result from the mental discipline such an education affords, he will find a familiar knowledge of Greek and Latin imperatively requisite, to enable him to understand the profession of surgery is a practical art, which cannot be acquired without continued personal intercourse with the sick; and this, they believe, can best be obtained by serving an *apprenticeship* to a regular practitioner, under whose inspection young men may not only prosecute their studies with the greatest advantage, but have frequent opportunities of being conversant with the sick, and of assisting in *preparing* and applying the means used for their recovery. The college further hope that every master will direct his apprentices as to the classes which they ought to attend, and the books which they ought to read; that he will occasionally subject them to examination, to ascertain their progress in the different branches of their education; and that he will explain the nature of the cases which they are attending under his superintendence.

the terms of art employed in medicine and its associated sciences, derived almost exclusively from those languages, and to comprehend, and also retain, the information imparted to him by his teachers. An acquaintance with the mathematical sciences, also, is scarcely less necessary, to enable the student to understand the admirable structure and functions of the human body, and to acquire habits of correct reasoning on the complicated phenomena of life and disease; and, since many valuable contributions to professional literature have been made in the French and German languages, it is desirable, when opportunity offers, or circumstances will permit, that he should likewise be instructed in those languages, so as to be enabled to read and translate them with facility. *During his apprenticeship,* a systematic course of study should be arranged, by which the pupil may be conducted progressively from elementary principles to the observance of disease; neither wasting his time by exclusive attention to practical pharmacy, nor employing it with as little profit in a premature attendance on the sick.

The society will not attempt, by any arguments, to give additional force to these strong recommendations of the college in favour of an apprenticeship, but merely request that it may be recollected, that the observations of the college, although they apply apparently to surgery only, are really meant for the surgeon-apothecary, or general practitioner; since in Scotland there is not any class of persons who devote themselves exclusively to the practice of surgery, as is the case in England, where the great mass of the profession are surgeon-apothecaries; the number who practise as surgeons only being very few, and of those who practise as apothecaries only, still fewer.

Although the society have not any intention of entering, on the present occasion, into the question of the length of time which may be usefully passed in an apprenticeship, they are nevertheless persuaded that many advantages attend this mode of beginning a professional life. It is not among the least of these advantages that a youth, at a very dangerous period of his life, is placed by his parents in a moral and religious family, and under the immediate eye and restraint, as well as instructions of a master, where he may acquire those habits of moral rectitude and self-control which he must practise in after-life, if he wishes to succeed in a profession which imperatively demands the strictest morality as the basis of its reputation. In favour of apprenticeships, too, it may be observed, that all the eminent surgeons of this country have been educated under that system, and that the College of Surgeons of London require an apprenticeship of not less than six years to be served by all persons who are educated under their immediate superintendence.

Lastly: the College of Surgeons of Edinburgh complain "that the power of the Society of Apothecaries is a monopoly;" that it is unjust to the licentiates of the college, as well as to others, who, like them, possess testimonials or diplomas from any of the different public bodies entrusted with the superintendence of medical education in these kingdoms; that it excludes from practice in England and Wales persons whose certificates of qualification are much more ample than those possessed by the licentiates of the society, and whose knowledge and acquirements are above all exception; and that it is injurious to the interests of the community."

The society deny that the Act of 55 Geo. III. gives them any "*monopoly*," and they are prepared to prove, that the objects which were contemplated, can only be carried into effect by the full, free, and independent exercise of the power which they possess under that Act. The society have always considered this power as a sacred trust, to be used for the benefit of the public only; and they feel that it would be impossible that the purposes of the trust could be accomplished, should the prayer of the petitioners be granted. It is not among the least important of their duties, that the society is by the Act required to enforce the penalties thereby imposed against persons who may practise illegally as apothecaries in England and Wales. This provision of the Act would be in a great measure defeated, should the licence granted by any other authority be rendered equivalent to that of the society; and in the ratio of the number of such authorities, would the power of enforcing the Act be diminished. It is already sufficiently difficult and expensive to enforce the law against ignorant and incompetent practitioners; and, should the alteration in the law now sought for be granted, it would become infinitely more difficult, as every authority empowered to grant the privilege of practising must on every occasion be referred to, for the purpose of ascertaining whether or not the particular individual complained of was licensed, and hence so much difficulty, uncertainty, and delay would result, that the Act could be enforced against few, if any persons, and the public would in this respect be placed in the same unprotected state in which they were previously to the passing of the Act.

In addition to these reasons, it is necessary also that the society should have independent control within their own jurisdiction, so that they may be enabled to prescribe the duration and extent of that education which they deem essential that candidates should possess; and not less requisite to enable the society to maintain, and gradually to improve the examination, which they may consider it indispensably necessary that every person should undergo who is to practise as an apothecary. The society do not deny the efficiency of the course of education prescribed by the College of Surgeons of Edinburgh, but they do not acknowledge that course to be better than their own; they contend, however, that if several authorities should be empowered to grant licences to practise within the same district, the standard of medical education and medical acquirements would be reduced to a very low scale, as it would tend to establish great inequality and laxity in the required education and examination; and students would naturally appear before that tribunal where they could obtain their licence to practise with the least expenditure of time, and with the smallest degree of mental application; and, should a student be rejected as incompetent by one authority, he would not re-appear before the same, but apply to another and another, until at last, finding one more lenient or less conscientious than the others, the incompetent person would obtain his licence, notwithstanding his previous rejections.

The College of Edinburgh state "That their diploma was admitted previously to 1815 as a qualification to practise surgery and pharmacy, and consequently to act as general practitioners of the healing art throughout the United Kingdom; and it has been, and is, received as a qualification in the medical services of the army and navy, and in the service of the Honourable East India Company." But this right of practising before 1815 did not belong to these persons in virtue of their diploma, as any person might then practise the healing art in England and Wales; and the statement, as far as regards the army, navy, and East India Company, is not correct; as all persons, whether holding the diploma of the college, or of any other body, are required, before entering those services, to be examined by the boards and medical officers appointed for that purpose, and the results of such examinations have not shown any superiority in the holders of Scottish diplomas.

In support of the assertion made by the College, that the course of study required by them is superior to that required by the society, they have annexed to their statement a comparative table, by the inspection of which they wish the public to draw an inference in their favour. This table, however, is fallacious, because it exhibits a partial view of the education of the general practitioner in

England. In Scotland every person not practising as a physician, is both a surgeon and an apothecary. The fellows of the College of Surgeons of Edinburgh practise in this double capacity, and the education required by the college is adapted for the practitioners of both surgery and medicine.

In England the Society of Apothecaries superintend the medical education alone of the general practitioner; who, in order to obtain a diploma in surgery, must, in addition to the medical studies required by the society, follow the course of education required by the College of Surgeons of London, and undergo examination in surgery by the Court of Examiners of that body, none of whom practise medicine. The English surgeon-apothecary thus passes examinations by two bodies, each preceded by appropriate studies, while the general practitioner in Scotland is examined by one body only.

The college claim for their licentiates and for the graduates of the Scotch universities a superiority in medical acquirements over the licentiates of the Society of Apothecaries. The society are, however, prepared to prove that this claim is not well founded; the records for the last year showing, that while one in six of candidates generally have been found unequal to pass the examination, one in three of these graduates and licentiates have failed.

For twelve months, commencing 1st May, 1832, and ending 25th April, 1833, the last day to which the statement is made up, the number stands thus:

| | Examined. | Rejected. | Passed. |
|---|---|---|---|
| Candidates in general | 364 | 57 | 307 |
| Graduates and licentiates of the Scottish colleges | 24 | 8 | 16 |

When it is considered that medicine is not an abstract science, but a practical art, it must be obvious that the student who has had the best opportunities of being practically educated will, *cæteris paribus*, be the best practitioner. In his own study, and in the lecture-room, the student may learn the principles of his profession; but it is at the bedside of the sick he must acquire that familiarity with disease which alone can make him an accomplished practitioner. It will avail but little that he is deeply read, and that he has attended most diligently at lectures, if he has not had the opportunity of being conversant with sickness and disease.

The following statement of the number of public hospitals and dispensaries will shew the superior opportunities of obtaining practical knowledge of the medical profession which London affords over the Scottish capital:

| EDINBURGH. | | LONDON. | |
|---|---|---|---|
| Number of students | 700 | Number of students | 1000 |
| Hospitals. | Patients. | Hospitals. | Patients. |
| The Royal Infirmary | 250 | St. Bartholomew's | 450 |
| Two dispensaries. | | St. Thomas's | 450 |
| | | Guy's | 540 |
| | | The London Hospital | 320 |
| | | St. George's | 250 |
| | | The Middlesex | 200 |
| | | The Westminster | 100 |
| | | Twelve or fourteen dispensaries. | |

The Infirmary of Edinburgh is so small, and the pupils so crowded, as to render it impossible for a tenth part of them to obtain a sight either of the patient or the visiting physician, nor can the majority even hear his report of the state of the patient or the remedies prescribed. Hence a practice has arisen of some person near to the physician repeating his observations and prescriptions verbally to those who are more distant. The report thus made is committed to writing by a few, and by the others, if heard at all, is probably soon forgotten; and it is obvious that little, if any, practical instruction can be derived from such means.

It requires, therefore, little observation to convince any uninterested and unprejudiced person how inadequate the opportunities of obtaining a practical knowledge of medicine are in Edinburgh, while in London, and many of the great towns in England, hospitals exist of so great an extent as to afford accommodation sufficient to enable double the number of students now attending to acquire a practical knowledge of their profession, all of which, in consequence of the education required by the Court of Examiners, have been converted into efficient schools of medicine. If, therefore, the most celebrated school in Scotland is so manifestly inferior to London in the opportunities which it affords of giving practical instruction, what can be said as to the ancient university of St. Andrew's, where there is no hospital, and but one professor to teach every branch of medical science?

The means, moreover, of teaching anatomy are, at Edinburgh, very insufficient, from the difficulty of procuring subjects, and the strong prejudices which exist against dissection; for, without a thorough knowledge of the structure of the human frame, it is impossible to acquire an accurate acquaintance with its diseases.

The foregoing statements, it is submitted, are sufficient to shew that the Act of Parliament was not obtained secretly and covertly, but was a measure demanded for the protection of the public against ignorant and uneducated persons, and that the conduct of the society was most open and unreserved; that the required apprenticeship has not the effect of preventing persons educated in Scotland from being examined for licenses to practise in England, and that the graduates and licentiates of the Scottish colleges do not possess attainments superior to the licentiates of the society. It is necessary, however, to disprove the statement made by the college, "that the power possessed by the society is injurious to the interests of the community." This statement is the only one in which the public is concerned, for the others have reference merely to the private interests of the Scottish colleges: and it will be most satisfactorily controverted by the statement of a few facts. Previously to the passing of the Act of Parliament, any person, whether educated or uneducated, and however ignorant he might be, had the right of practising the profession of medicine in England; and, although the greater number of medical practitioners were not so fully educated as it was desirable they should be, there were however to be found among them many possessing qualifications of the highest order. These differences in professional attainments resulted from the non-existence of any authority either to direct the education, or to ascertain the competency of persons to practise their profession.

The education of the great majority of students who at that time resorted to London, for the purpose, as it was then popularly called, of "walking the hospitals," was extremely defective, their attention being chiefly, if not exclusively, directed to surgery; the course of study being confined to an attendance on one or two courses of lectures on anatomy, and one on surgery; and for a few months they gave a desultory and superficial attendance on the surgeon's practice of an hospital; a few attended lectures on the practice of medicine, and still fewer lectures on chemistry; but materia medica and therapeutics were almost wholly, and the medical, or physician's practice at the hospitals was entirely, neglected. A course of study so little calculated to fit students for the duties of their profession required alteration; and the Court of Examiners of the society applied themselves to the performance of this very important duty with zeal and discretion. They were well qualified to superintend the education of persons destined to practise the same branch of the profession which they themselves practised: and, although fully sensible that medical education was susceptible of much improvement, they were, nevertheless, unwilling to make any extensive alterations precipitately; the increase of study required has consequently been progressive: and although the Examiners consider that their regulations are yet capable of further improvement, yet they feel assured that the plan of medical education laid down by them is, in every respect, equal to that of any university or college in any part of Great Britain. The power vested in the Court of Examiners by that clause of the Act which authorizes them to require of all candidates for examination "testimonials of a sufficient medical education," has enabled them to require and enforce the present course of study, in which it will be perceived that every branch of science which has any relation to the practice of medicine is included; and that the valuable field of information afforded by the great hospitals of England cannot, as formerly, be neglected.

In proof of this fact, it is only necessary to refer to the following table of the number of pupils who attended the physician's practice at the several hospitals in London, in the year 1814 (the year before the passing of the Act), and in the year 1832.

| Hospitals. | Year 1814. | Year 1832. |
| --- | --- | --- |
| St. Bartholomew's | 6 | 87 |
| St. Thomas's | 1 | 86 |
| Guy's | 16 | 62 |
| London | 0 | 20 |
| Middlesex | 1 | 32 |
| St. George's | 14 | 31 |
| Westminster | 0 | 8 |

The effects of the impulse which was given to medical education in England by these progressive demands of the court for a longer and more extensive course of study, are clearly demonstrated by the better organization of the medical schools of London, and by the establishment of medical schools in many of the principal towns of England, viz. Bath, Bristol, Birmingham, Manchester, Leeds, Hull, Liverpool, and Sheffield, in which all the branches of medicine are taught by gentlemen of undoubted competence. The same consideration which induced the Court of Examiners not to make a sudden and extensive alteration in the course of study, induced them also to make their examinations during the first few years less extensive than they have since become, but such examinations have always embraced every branch of science connected with the practice of medicine. The following table exhibits the results of these examinations for every year since the establishment of the Court of Examiners:

| From August 1st to July 31st. | Examined. | Rejected. | Passed. |
| --- | --- | --- | --- |
| 1815 to 1816 | 185 | 12 | 173 |
| 1816 — 1817 | 192 | 11 | 181 |
| 1817 — 1818 | 226 | 17 | 209 |
| 1818 — 1819 | 268 | 15 | 253 |
| 1819 — 1820 | 275 | 20 | 255 |
| 1820 — 1821 | 297 | 13 | 284 |
| 1821 — 1822 | 340 | 20 | 320 |
| 1822 — 1823 | 409 | 28 | 381 |
| 1823 — 1824 | 374 | 24 | 350 |
| 1824 — 1325 | 397 | 32 | 365 |
| 1825 — 1826 | 488 | 43 | 445 |
| 1826 — 1827 | 465 | 47 | 418 |
| 1827 — 1828 | 510 | 70 | 440 |
| 1828 — 1829 | 365 | 65 | 300 |
| 1829 — 1830 | 525 | 86 | 439 |
| 1830 — 1831 | 465 | 104 | 361 |
| 1831 — 1832 | 446 | 73 | 373 |

The name of each rejected candidate, together with the causes of his failure, are entered in a book at the time of his rejection: these books the society will be quite ready to produce, whenever either House of Parliament, or his Majesty's Government, may be pleased to call for them.

The Court of Examiners have also established a system of registration, by which the attendance of the student on all the courses of lectures required by the court is insured, and by which they have perfect knowledge of the present standing of every medical student in England, as to the particular lectures which he is attending, and at what school. The benefit of these arrangements is apparent in the improved character of medical students; and the teachers in the different schools bear ample testimony to their increased diligence and zeal in the acquirement of knowledge; and all persons who take an interest in the subject unhesitatingly acknowledge that the society has done more for the advancement of medical education, and for the benefit of the community, than any other medical authority in the united kingdom.

The society is prepared to show that the sums of money received by them for certificates of qualification, are not more than sufficient to defray the unavoidable charges of enforcing the law against incompetent and unqualified practitioners, and the other necessary expenses of administering the Act; and that no part whatever of the sums so received has been, or is, appropriated to the private purposes of the society.

The examiners are not permitted to be lecturers, or to have any interest whatever in any medical school; so that no temptation exists to induce them, at their examinations, to shew favour or affection to candidates; for, not being connected with any school or hospital, they are consequently in a situation to be perfectly impartial; while, in Scotland, the professors at the different universities are the examiners of their own pupils.

The society, in concluding this reply, have only to add, that they have always, to the best of their abilities, and in the honest exercise of their judgment, endeavoured faithfully and conscientiously to perform all the duties entrusted to them; that the successive Courts of Examiners have established a competent course of education, and an efficient examination; and that they have ever shewn that they considered themselves as invested with a sacred and responsible power, to be used for the public good, and for that alone. They therefore submit, that the statement made by the College of Surgeons of Edinburgh, "that the present state of the law is injurious to the interests of the community," has been fully disproved; and that it is apparent that the Act of Parliament of which they complain has been eminently conducive to the welfare of the public, and ought not to be altered without the most deliberate and unprejudiced inquiry into the whole subject; and the Society of Apothecaries declare their perfect readiness to meet such inquiry, whenever Parliament shall think fit to entertain the question.

---

*The following are the Regulations of the Court of Examiners of the Society of Apothecaries, to be observed by Candidates for their Certificate.*

Every candidate for a certificate to practise as an apothecary will be required to produce testimonials,

Of having served an apprenticeship of not less than five years to an apothecary, as required by the fifteenth clause of the Act for the better regulating the Practice of Apothecaries. Of having attained the full age of twenty-one years; and of good moral conduct.

Students whose attendance on lectures commenced on or after January 1831, must, in addition to these testimonials, adduce proof of having devoted at least two years to an attendance on lectures and hospital-practice, and of having attended the following courses of lectures:

Chemistry, two courses; each course consisting of not less than forty-five lectures.

Materia Medica and Therapeutics, two courses; each course consisting of not less than forty-five lectures.

Anatomy and Physiology, two courses; Anatomical Demonstration, two courses; of the same extent as required by the Royal College of Surgeons of London.

Principles and Practice of Medicine, two courses; each course consisting of not less than forty-five lectures. To be attended subsequently to the termination of the first course of lectures on chemistry, materia medica, and anatomy and physiology.

Botany, one course, consisting of not less than thirty lectures; to be attended between the 1st of April and 31st of October.

Midwifery, and the Diseases of Women and Children, two courses.

Forensic Medicine, one course; to be attended during the second year.

Students are likewise earnestly recommended to avail themselves of instruction in Morbid Anatomy.

The candidate must also have attended for twelve months, at least, the physician's practice at an hospital containing not less than sixty beds, and where a course of clinical lectures is given; or for fifteen months at an hospital wherein clinical lectures are not given; or for fifteen months at a dispensary connected with some medical school recognized by the court. No part of this attendance can be entered upon until the termination of one entire year from the commencement of attendance on lectures, nor until one course of lectures, at least, on chemistry, materia medica, anatomy, and the practice of medicine, have been attended, in the order prescribed by the regulations.

The examination of the candidate for a certificate of qualification to practise as an apothecary is as follows:

1. In translating parts of Celsus de Medicinâ, or Gregory's Conspectus Medicinæ Theoreticæ, physician's prescriptions, and the Pharmacopœia Londinensis; 2, in Chemistry; 3, in Materia Medica and Therapeutics; 4, in Botany; 5, in Anatomy and Physiology; 6, in the Principles and Practice of Medicine. (This branch of the examination embraces an inquiry into the diseases of pregnant and puerperal women, and also into the diseases of children.)

---

### LITERARY INTELLIGENCE.

Shortly will be published, (dedicated, by permission, to the King,) a History of Mummies, by THOMAS JOSEPH PETTIGREW, F.R.S. F.A.S. F.L.S., Member of the Royal Asiatic Society, Doctor of Philosophy of the University of Gottingen, Surgeon to the Charing-cross Hospital, Asylum for Female Orphans, &c. &c.

The object of this work will be to give a complete and entire history of mummies, human and comparative, both natural and artificial, from the earliest period to the present time; to detail the various processes of embalming adopted by the ancients; and to treat the subject as regards its antiquity, its relation to the natural history of man, to natural history in general (in reference to the substances used to prevent putrefaction from taking place;) the state of the arts and manufactures among the ancient Egyptians, as shewn in the structure of the cases, bandages, &c.; also to give an account of the various idols, emblems, coins, inscriptions, papyri, &c., that have been found enclosed in mummies, and of the different methods that have been practised in modern times to imitate the processes of the ancients. The whole will be illustrated by numerous plates, representing mummies of all kinds in their several states and conditions, and the substances which have been met with in the unfolding of them.

---

### MONTHLY LIST OF MEDICAL BOOKS.

### METEOROLOGICAL REGISTER,

By Messrs. Harris and Co., Mathematical Instrument Makers, 50, High Holborn.

| | Moon. | Rain gauge. | Thermometer | | | Barometer. | | De Luc's Hygrometer | | Winds. | | Atmospheric Variation. | | |
|---|---|---|---|---|---|---|---|---|---|---|---|---|---|---|
| May | | | 9 a.m. | max. | min. | 9a.m. | 10p.m. | 9a.m. | 10p.m. | 9 a.m. | 10p.m. | 9 a.m. | 2 p.m. | 10 p.m. |
| 20 | | | 60 | 66 | 55 | 29.90 | 29.92 | 76 | 74 | WNW | WNW | cloudy | fine | cloudy |
| 21 | | | 62 | 70 | 56 | 30.14 | 30.14 | 70 | 69 | WNW | S | fine | | |
| 22 | | | 65 | 70 | 55 | .15 | .15 | 67 | 65 | SE | SE | | | |
| 23 | | | 65 | 68 | 55 | .15 | .10 | 65 | 63 | ESE | SE | | | |
| 24 | | | 68 | 74 | 57 | .08 | 29.99 | 63 | 60 | ESE | SSE | | | |
| 25 | | | 66 | 76 | 55 | 29.83 | .83 | 58 | 57 | WSW | ENE | | | |
| 26 | ☽ | | 61 | 67 | 48 | .98 | 30.83 | 57 | 57 | E | NE | | | |
| 27 | | | 55 | 62 | 50 | 30.08 | .03 | 57 | 58 | ESE | ENE | | | |
| 28 | | | 60 | 69 | 52 | .03 | 29.93 | 58 | 57 | WNW | NE | | | |
| 29 | | | 61 | 64 | 51 | 29.93 | .94 | 58 | 58 | E | SE | | | |
| 30 | | | 61 | 62 | 48 | 30.03 | 30.01 | 57 | 59 | ESE | SE | | | |
| 31 | | | 60 | 66 | 52 | .01 | 29.95 | 60 | 59 | SE | SE | | | |
| June | | | | | | | | | | | | | | |
| 1 | | | 70 | 74 | 57 | 29.82 | .73 | 59 | 54 | S | SW | | | cloudy |
| 2 | ○ | | 63 | 70 | 55 | .58 | .33 | 56 | 62 | WSW | WSW | | | rain |
| 3 | | | 61 | 65 | 52 | .28 | .37 | 59 | 59 | W | SW | | | fine |
| 4 | | | 60 | 65 | 53 | .37 | .41 | 59 | 59 | SSW | WSW | | | cloudy |
| 5 | | | 61 | 67 | 54 | .46 | .56 | 59 | 58 | WNW | WSW | | | cloudy |
| 6 | | | 66 | 73 | 60 | .57 | .63 | 58 | 58 | SSW | WSW | | | |
| 7 | | | 68 | 73 | 60 | .77 | .83 | 58 | 59 | NW | WNW | cloudy | fine | fine |
| 8 | | | 68 | 71 | 62 | .93 | 30.02 | 59 | 57 | NW | NW | | | |
| 9 | | | 71 | 75 | 55 | 30.04 | .04 | 58 | 58 | NW | NW | fine | | |
| 10 | ☾ | | 63 | 76 | 63 | .00 | 29.85 | 60 | 60 | NW | S | | | |
| 11 | | | 69 | 71 | 54 | 29.46 | .55 | 59 | 58 | WSW | W | cloudy | | cloudy |
| 12 | | | 60 | 63 | 54 | .52 | .44 | 58 | 60 | NW | W | | | |
| 13 | | | 57 | 63 | 53 | .40 | .25 | 61 | 67 | WSW | WSW | rain | | cloudy |
| 14 | | .25 | 61 | 63 | 48 | .20 | .43 | 65 | 63 | NW | ENE | cloudy | | fine |
| 15 | | | 57 | 65 | 55 | .63 | 67 | 62 | 63 | ENE | SSW | fine | | |
| 16 | | | 61 | 68 | 58 | .44 | .58 | 68 | 67 | S | SW | rain | cloudy | fine |
| 17 | ● | .28 | 65 | 70 | 55 | .62 | .69 | 65 | 66 | W | W | fine | fine | |
| 18 | | .15 | 65 | 70 | 58 | .84 | .87 | 63 | 62 | W | WSW | cloudy | | |
| 19 | | | 65 | 69 | 60 | .81 | .75 | 62 | 64 | WSW | SW | | rain | showery |

The quantity of Rain fallen in May, was 78.100ths of an inch.

